# Age as the Impact on Mortality Rate in Trauma Patients

**DOI:** 10.1155/2022/2860888

**Published:** 2022-10-26

**Authors:** Onchuda Wongweerakit, Osaree Akaraborworn, Burapat Sangthong, Komet Thongkhao

**Affiliations:** Division of Trauma and Critical Care, Department of Surgery, Faculty of Medicine, Prince of Songkla University, Hat Yai, Songkhla, Thailand

## Abstract

**Background:**

Globally, the fastest-growing population is that of older adults. Geriatric trauma patients pose a unique challenge to trauma teams because the aging process reduces their physiologic reserve. To date, no agreed-upon definition exists for the geriatric trauma patients, and the appropriate age cut point to consider patients at increased risk of mortality is unclear.

**Objectives:**

To determine the age cut point at which age impacts the mortality rate in trauma patients in Thailand.

**Materials and Methods:**

This was a retrospective cohort and prognostic analysis study conducted in trauma patients ≥40 years. Patient data were retrieved from the trauma registry database and hospital information system in Songklanagarind Hospital. The estimated sample size of 1,509 patients was calculated based on the trauma registry data. The age with the maximum mortality rate was used as the cut point to define the elderly population. Hospital cost, intensive care unit (ICU) length of stay, gender, precomorbidity, mechanism of injury, injury severity score (ISS), and trauma and injury severity score were analyzed for any correlation with mortality, and whether or not they were associated with elderly trauma patients.

**Results:**

A total of 1,523 trauma patients ≥40 years were included in the study. The median age in both the survival and death groups was 61 years, with gender in both groups being similar (*p* value = 0.259). In the multivariate logistic regression analyses, the adjusted odds ratio (OR) showed that increasing age was significantly associated with mortality (OR = 1.05; 95% CI, 1.02–1.07; *p* value <0.001). In the age group of 70 to 79 years and >80 years, the odds of mortality were significantly increased (OR 3.29, 95% CI, 1.24–8.68; *p* value = 0.016 and OR 3.29, 95% CI, 1.27–12.24; *p* value = 0.018, respectively).

**Conclusion:**

Age is a significant risk factor for mortality in trauma patients. The mortality significantly increased at the age of 70 and higher.

## 1. Introduction

Globally, the fastest growing population consists of older adults, with the rate of all aging populations growing much faster than in the past. The proportion of the world's population aged over 60 will increase by almost double from 12% to 22% between 2015 and 2050, as reported by WHO. The number of people aged 60 and over in Thailand stands accounting for 20% of the population. By 2050, Thailand's aging population is expected to increase to 35.8% of the population [1].

In the United States, 25% of the total annual trauma admissions are elderly patients [2]. In 2018, data from death certificate found that injury is the fifth leading cause of death in Thailand [3]. Geriatric trauma patients, in the context of older aged patients in trauma act a particular challenge to trauma teams because the aging process diminishes their physiologic reserve to respond to shock and injury as well as their idiosyncratic pulmonary and cardiovascular responses [4, 5]. Although the mechanisms of injury are in the same way as the younger population, mortality is higher in older adults. For these reasons, failure to properly triage older trauma patients may in part contribute to mortality.

The definition of geriatric trauma is unclear [6]. The American College of Surgeons Advanced Trauma Life Support (ATLS) Course (found on the mid-1980s) suggests 55 years of age as the criterion to consider transporting trauma patient to a trauma center [7]. In spite of the ATLS recommendations, the optimal cut-off point of age to identify patients at increased risk of mortality is still unclear. A literature review of 20 years found that advancing age was associated with higher mortality rates, ranging from 45 to 84 years [8–23]. Other reported factors [5, 12, 13, 16, 17] that impacted mortality and morbidity in trauma patients were gender, precomorbidity, injury severity score (ISS), trauma injury severity score (TRISS), and mechanism of injury. A study from Holcomb et al. [23] show that physiologic changes started at an age of 45 years. This study aimed to determine the specific age at which increased mortality was observed in trauma. Acknowledgment research was funded by Targeted Research Grants, Faculty of Medicine, Prince of Songkla University.

## 2. Methods

### 2.1. Data Source and Patient Selection

This was a retrospective cohort study of trauma patients, obtained from the hospital information system and trauma registry database of Songklanagarind Hospital, which is a Level 1 trauma center in Southern Thailand. All records of patients aged ≥40 years and admitted between January 2016 and December 2019 were reviewed. Patients who died before arrival were excluded. This project was approved by the Human Research Ethics Committee Prince of Songkla University (REC.61-147-10-4).

The estimated sample size was calculated based on the trauma registry data, to achieve 90% power at a 5% significance level (one sided). Logistic regression was used to control the confounders, with the hypothesis that each year of increasing age impacted the log odds of mortality. Finally, the estimated sample size was 1,509.

### 2.2. Patient Variables

The primary outcome of this study was the age cut point to having an impact on mortality in trauma patients. Characteristics of the patients included age, gender, precomorbidities, and the injury details (i.e., ISS, TRISS, and mechanism of injury). The outcomes of treatment were identified as mortality rate, intensive care unit (ICU) length of stay, and hospital cost. Precomorbidities, including, diabetic mellitus, ischemic heart disease, malignancy, cirrhosis, renal disease/kidney stone, coagulopathy, chronic obstructive pulmonary disease/asthma, cerebrovascular accident or transient ischemic attack, acquired immunodeficiency syndrome, hypertension, and dyslipidemia, were reviewed for any association with mortality.

### 2.3. Statistical Analysis

Mean and standard (SD) and median and interquartile range (IQR) were calculated for continuous variables. The percentage was calculated for categorical variables. Univariate analyses of continuous and categorical variables were performed with the Wilcoxon rank-sum (Mann–Whitney) test and Pearson chi-square test, as appropriate. Statistical significance was determined at *p* value <0.05. We performed multivariate logistic regression to assess the association between age and the risk of mortality, and adjusted odds ratios (OR) with 95% confidence intervals (CI), after adjusting for gender and ISS. All statistical analyses were conducted with STATA, version 15 software.

## 3. Results

From January 2016 to December 31, 2019, 1,552 records of trauma patients aged 40 years and above were eligible for the study. After exclusion of 29 patients, who died before arrival at the hospital, the number of eligible patients was 1,523. The survival group had 1,464 patients (96%), and the death group had 59 patients (4%). The characteristics of the study population as well as associated factors are presented in Table 1. Comparisons between the survival and death groups showed that the median age in both the groups was 61 years, with precomorbidities being similar in both the groups, with the exception of ischemic heart disease and renal disease/kidney stone which were higher in the death group. The median ISS in the death group was higher than the survival group (29 vs. 9, *p* value <0.001). The median ICU length of stay was greater in the death group than in the survival group (2 vs. 0 days, *p* value <0.001). The median (IQR) hospital cost in the death group was higher than the survival group (100,300 Thai baht or 3380 USD (42,449–202,042 Thai baht or 1431–6809 USD) vs. 54,153 Thai baht or 1825 USD (23,014–133,778 Thai baht or 776–4508 USD); *p* value <0.001).

In the multivariate logistic regression analyses adjusted for gender, ISS, systolic blood pressure (SBP), pulse rate (PR), and precomorbidities, the adjusted OR for mortality of age was 1.05 (95% CI, 1.02–1.07; *p* value <0.001) (Table 2).

Age was categorized into 10-year intervals. The characteristics of patients that were stratified by age groups are shown in Table 3. The mortality rate was adjusted by gender, ISS, systolic blood pressure (SBP), pulse rate (PR), and precomorbidities to determine the cutoff age with the highest mortality risk. In the age group of 70 to 79 years and >80 years, the odds of mortality were significantly increased (OR 3.29 95% CI, 1.24–8.68; *p* value = 0.016 and OR 3.90 95% CI, 1.27–12.24; *p* value = 0.018, respectively) (Table 4).

## 4. Discussion

Geriatric trauma lacks a uniformed age criterion to define an elderly trauma patient [6, 8, 24] with the literature defining the ages of patients to be ranging between 45 and 84 years [8–23]. This study collected data from the age of 40 and found the age above 70 significantly increased the mortality rate after adjusted gender, ISS, systolic blood pressure (SBP), pulse rate (PR), and precomorbidities. Therefore, we suggest that all trauma patients older than 70 years should receive more vigilant care during admission. The studies of Goodmanson et al. reported age as a continuous variable and found the cut point was 57 and the age above 74 has not increased the mortality rate [13]. Another continuous data report of Hashmi A et al., however, found the cut points of age that increase mortality rate was 75 years [9]. We believe each country should have its cut point since we are different in characters of population. The population in Thailand has a life expectancy of 77 years, [25] and the health conditions of the elderly in Thailand should be different from Japan where the life expectancy of the population is 84 years [26]. Besides the differences in life expectancy, the health condition in Thailand is also different than other countries. A study from Thinuan et al. [27] performed a cross-sectional study to investigate the frailty in the community and found that Thailand had prevalence of frailty as 13.9% and robustness at 50.9%. Compared with a study from Kojima et al. [28] that conducted a systematic review and meta-analysis on prevalence of frailty in Japan, only 7.4% of the population was frail and 44.4% was robustness.

The death rate in elderly trauma patients also showed significant differences across the countries. The death rate of patients older than 70 years in our study was 4.4%. A study from Lee et al. [29] that reported the in-hospital mortality rate of elderly trauma patients in Korea defined elderly as age greater than 65 years and the mortality rate was 1%. On the other hand, a study from Gera et al. [30] reported the mortality rate of trauma patients who were older than 60 years and found the mortality rate was 23.6%.

Elderly trauma usually comes with premorbidities. In our study, almost half of the population had precomorbidity. The precomorbidities in this study that were associated with mortality were ischemic heart disease and renal disease/kidney stone. A study by Bradburn E et al. [5] had the same results as our study. We anticipate that the patients cannot respond to aggressive resuscitation due to decreased pulmonary functions and cardiovascular response to injury. Adams S. D. et al. [22] analyzed morbidity after injury in elderly trauma patients and found that the complications related to single end-organ failure after injury were renal, pulmonary, and cardiac complications. Therefore, elderly trauma patients with ischemic heart disease and renal disease/kidney stone should receive more attention.

## 5. Limitations

This study had some limitations. This was a single-center retrospective review; therefore, the number of patients in this study was lower than in previous studies. However, this was the first study conducted in South East Asia. Since each country has different life expectancies due to the differences in healthcare systems, the results of this study can be generalized to other countries which have the same characteristics of this population. The results of this study can be used to benefit our hospital to establish an age cut point to triage older trauma patients into a priority group, so as to improve the survival rate in elderly trauma patients.

## 6. Conclusion

Our results showed that older trauma patients had a significantly increased risk of mortality beginning at the age of 70 years. An age of 70 years could be considered an appropriate cut off for considering and developing geriatric trauma triage criteria in our institute. The future multicenter research is needed to establish the national triage criteria.

## Figures and Tables

**Table 1:** Characteristics of patients compared between the survival group and death group.

Variables	Survival (*n* = 1,464)	Death (*n* = 59)	*p* value
Age, years, median (IQR)	61 (50–74)	61 (52–77)	0.3294
Gender			0.259
Male	937 (64)	47 (71)	

*Precomorbidities*, *n (*%)			
Diabetic	203 (43.1)	8 (57.1)	0.3
Ischemic heart disease	73 (20.17)	5 (45.45)	0.042
Malignancy	42 (13.9)	1 (14.2)	0.88
Cirrhosis	18 (5.7)	0 (0)	0.547
Renal disease/kidney stone	52 (15.2)	6 (50)	0.001
Coagulopathy	20 (6.23)	1 (14.2)	0.389
COPD/asthma	58 (16.8)	1 (14.2)	0.859
CVA or TIA	85 (23.1)	4 (40)	0.216

*Precomorbidities*, *n* (%)			
AIDS	9 (2.9)	0 (0)	0.671
Hypertension	436 (66.6)	17 (85)	0.085
DLP	257 (50.6)	8 (57.1)	0.634
Other	417 (66.5)	19 (76)	0.323
Unknown	115 (29)	21 (80.7)	<0.001

ISS, median (IQR)	9 (5–16)	29 (22–34)	<0.001
TIRSS, median (IQR)	0.9805 (0.9655–0.9928)	0.662 (0.2215–0.8836)	<0.001
Mechanism of injury, *n* (%)			0.031
Motor vehicle collision	127 (8.7)	3 (5.1)	
Gunshot wound (GSW)	31 (2.1)	3 (5.1)	
Fall from height	68 (4.6)	3 (5.1)	
Stab wound (SW)	20 (1.4)	0	
Ground-level falls	473 (32.3)	15 (25.4)	
Pedestrian	45 (3.1)	0	
Motorcycle/bicycle accident	518 (35.4)	33 (55.9)	
Blast injury	18 (1.2)	0	
Burn	14 (0.9)	1 (1.7)	
Other	150 (10.3)	1 (1.7)	
ICU stay, days, median (IQR)	0	2 (0–4)	<0.001
Hospital cost, USD, median (IQR)	1825 (776–4508)	3380 (1431–6809)	<0.001

Note: IQR, interquartile range; COPD, chronic obstructive pulmonary disease; CVA, cerebrovascular accident; TIA, transient ischemic attack; AIDS, acquired immunodeficiency syndrome; DLP, dyslipidemia; ISS, injury severity score; TRISS, trauma and injury severity score; ICU, intensive care unit.

**Table 2:** Adjusted odds ratio for mortality as a function of age.

Outcomes	Odds ratio	95% CI	*p* values
Age	1.05	1.02–1.07	<0.001
Gender	0.85	0.44–1.65	0.629
ISS	1.14	1.10–1.17	<0.001
SBP	0.99	0.98–0.99	0.004
PR	1.0	0.99–1.01	0.314
Precomorbidities	0.69	0.34–1.41	0.314

Note: CI, confidence interval; ISS, injury severity score; SBP, systolic blood pressure; PR, pulse rate.

**Table 3:** Characteristics of patients stratified by age groups.

	40–49	50–59	60–69	70–79	>80
Number of patients, *n*	355	352	317	249	250
Gender male, *n* (%)	254 (71.6)	236 (67.1)	210 (66.3)	146 (58.3)	133 (53.2)
Mechanism of injury, *n* (%)					
Motor vehicle collision	48 (13.5)	39 (11.1)	29 (9.2)	11 (4.5)	3 (1.2)
Gunshot wound (GSW)	13 (3.7)	13 (3.7)	7 (2.2)	1 (0.4)	0 (0)
Fall from height	22 (6.2)	15 (4.3)	19 (5.9)	12 (4.8)	3 (1.2)
Stab wound (SW)	7 (1.9)	6 (1.7)	4 (1.3)	1 (0.4)	2 (0.8)
Ground-level falls	26 (7.3)	45 (12.8)	84 (26.5)	136 (54.6)	197 (78.8)
Pedestrian	5 (1.4)	11 (3.1)	15 (4.7)	10 (4.0)	4 (1.6)
Motorcycle/bicycle accident	160 (45.1)	170 (48.3)	132 (41.6)	63 (25.3)	26 (10.4)
Blast injury	14 (3.9)	4 (1.1)	0 (0)	0 (0)	0 (0)
Burn	3 (0.9)	6 (1.7)	4 (1.3)	0 (0)	2 (0.8)
Other	57 (16.1)	43 (12.2)	23 (7.3)	15 (6.0)	13 (5.2)
Initial SBP, mmHg, mean (SD)	134 (29)	140 (30)	146 (30)	148 (30)	151 (29)
Initial PR, bpm, mean (SD)	87 (21)	86 (21)	82 (17)	82 (18)	83 (18)
ISS, median, IQR	10 (5–17)	10 (5–17)	9 (5–17)	9 (5–16)	9 (5–11)
TRISS, median, IQR	0.9955 (0.9890–0.9973)	0.9824 (0.9629–0.9958)	0.9724 (0.9537–0.9811)	0.974 (0.9537–0.9811)	0.977 (0.9629–0.9796)

Note: IQR, interquartile range; ISS, injury severity score; TRISS, trauma and injury severity score; ICU, intensive care unit.

**Table 4:** Stratified age for confidence interval.

Age group	Odds ratio	95% CI	*p* values
40–49	References		
50–59	1.40	0.57–3.46	0.463
60–69	1.42	0.50–4.00	0.509
70–79	3.29	1.24–8.68	0.016
>80	3.90	1.27–12.24	0.018

Note: CI, confidence interval.

## Data Availability

The data used to support the findings of this study are available from the corresponding author upon request.
